# Tolerability and efficacy of durvalumab, either as monotherapy or in combination with tremelimumab, in patients from Asia with advanced biliary tract, esophageal, or head‐and‐neck cancer

**DOI:** 10.1002/cam4.4593

**Published:** 2022-05-24

**Authors:** Yuichiro Doki, Makoto Ueno, Chih‐Hung Hsu, Do‐Youn Oh, Keunchil Park, Noboru Yamamoto, Tatsuya Ioka, Hiroki Hara, Manabu Hayama, Masahiro Nii, Keiko Komuro, Mariko Sugimoto, Makoto Tahara

**Affiliations:** ^1^ Osaka University Hospital Osaka Japan; ^2^ Kanagawa Cancer Center Yokohama Japan; ^3^ National Taiwan University Hospital and National Taiwan University Cancer Center Taipei City Taiwan; ^4^ Department of Internal Medicine Seoul National University Hospital, Cancer Research Institute, Seoul National University College of Medicine Seoul Republic of Korea; ^5^ Samsung Medical Center Sungkyunkwan University School of Medicine Seoul Republic of Korea; ^6^ National Cancer Center Hospital Tokyo Japan; ^7^ Osaka International Cancer Institute Osaka Japan; ^8^ Saitama Cancer Center Saitama Japan; ^9^ AstraZeneca Osaka Japan; ^10^ National Cancer Center Hospital East Kashiwa Japan

## Abstract

**Background:**

Agents targeting the programmed cell death‐1 pathway have demonstrated encouraging activity across multiple solid tumor types. The dose expansion phase of this phase I study evaluated the safety, tolerability, and antitumor activity of durvalumab monotherapy, and durvalumab plus tremelimumab (an anti‐cytotoxic T‐lymphocyte‐associated antigen 4 monoclonal antibody) combination therapy, in patients from Asia with biliary tract cancer (BTC), esophageal squamous cell carcinoma (ESCC), or head and neck squamous cell carcinoma (HNSCC).

**Methods:**

Patients with advanced BTC, ESCC, or HNSCC with disease progression during or following ≥1 platinum‐based therapy received durvalumab monotherapy (10 mg/kg every 2 weeks) or durvalumab plus tremelimumab (durvalumab 20 mg/kg every 4 weeks [Q4W] plus tremelimumab 1 mg/kg Q4W for 4 doses, followed by durvalumab 20 mg/kg Q4W). The primary objective was safety and tolerability. Secondary objectives included antitumor activity.

**Results:**

Durvalumab monotherapy was assessed in 116 patients (median age 63.5 years, 75.9% male) of whom, 42, 42, and 32 had BTC, ESCC, or HNSCC, respectively. Grade ≥3 treatment‐related adverse events (TRAEs) were reported in 19.0%, 9.5%, and 25.0% of patients with BTC, ESCC, and HNSCC, respectively. Objective response rate (ORR) was 4.8%, 7.1%, and 9.4% in BTC, ESCC, and HNSCC. Durvalumab plus tremelimumab was evaluated in 124 patients (median age 62.0 years, 79.8% male) of whom 65 had BTC and 59 had ESCC. Grade ≥3 TRAEs were reported in 23.1% and 13.6% of patients with BTC and ESCC. ORR was 10.8% and 20.3% in BTC and ESCC. There were two complete responses and 10 partial responses in ESCC, and seven partial responses in BTC.

**Conclusion:**

In general, durvalumab monotherapy and durvalumab plus tremelimumab combination therapy displayed acceptable safety profiles consistent with published literature, and also demonstrated clinical benefit, in patients from Asia with BTC, ESCC, or HNSCC with disease progression on ≥1 prior treatment. ClinicalTrials.gov Identifier: NCT01938612.

## Graphical abstract

Durvalumab monotherapy and durvalumab plus tremelimumab both showed manageable toxicity profiles and clinical benefit in Asian patients with BTC, ESCC, and HNSCC who had progressed on previous systemic chemotherapy.

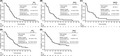

## 1 INTRODUCTION

The principles underlying programmed cell death‐1 (PD‐1) or programmed death ligand‐1 (PD‐L1) inhibition as a cancer immunotherapy are well established,^1^ and single agents targeting this pathway have demonstrated encouraging activity across multiple solid tumor types.^1^, ^2^ The efficacy and safety of durvalumab (anti‐PD‐L1 monoclonal antibody [mAb]) have been demonstrated in large, international studies of patients with advanced/metastatic non‐small‐cell lung cancer (NSCLC),^3^, ^4^ extensive‐stage small‐cell lung cancer,^5^ and urothelial carcinoma.^6^, ^7^ Following these recent successes across solid tumors and lines of therapy, evaluating checkpoint inhibitors in other malignancies, especially those with high unmet need, could demonstrate potential to improve upon standard of care, especially in tumors thought to be highly immunogenic.

Preclinical data and preliminary clinical studies suggest encouraging mechanistic effects for immunotherapy in biliary tract cancers (BTC), including intrahepatic and extrahepatic cholangiocarcinoma, gallbladder cancer, and ampullary carcinoma subtypes.^8^, ^9^, ^10^ In esophageal squamous cell carcinoma (ESCC), agents targeting the PD‐1/PD‐L1 pathway have demonstrated encouraging activity; nivolumab is approved in the United States for patients with unresectable advanced, recurrent, or metastatic ESCC after prior fluoropyrimidine and platinum‐based chemotherapy^11^; pembrolizumab is approved for patients with recurrent locally advanced or metastatic ESCC with disease progression after ≥1 prior line of systemic therapy and whose tumors express high PD‐L1.^12^ PD‐1/PD‐L1 mAbs are approved for the treatment of platinum‐refractory recurrent or metastatic (R/M) head and neck squamous cell carcinoma (HNSCC).^12^ Furthermore, the US Food and Drug Administration (FDA) has approved pembrolizumab in combination with platinum and fluorouracil for all patients with HNSCC, and as monotherapy for patients with PD‐L1‐positive disease as first‐line treatment for metastatic or unresectable, recurrent HNSCC. US FDA approval has also been granted for pembrolizumab monotherapy for all patients with R/M HNSCC and disease progression during or after platinum‐containing chemotherapy.^12^, ^13^ Consistent with the EAGLE (phase III), HAWK, and CONDOR (both phase II) studies, durvalumab demonstrated antitumor activity in R/M HNSCC.^14^, ^15^, ^16^

Tremelimumab (a mAb that blocks cytotoxic T‐lymphocyte‐associated antigen 4 [CTLA‐4]) may provide additive or synergistic effects in combination with anti–PD‐1/PD‐L1 therapy.^2^ Accordingly, combined anti–PD‐L1/anti–CTLA‐4 therapy has been shown to be effective across multiple tumor types, as initially confirmed in advanced melanoma^17^, ^18^, ^19^ and also in NSCLC^20^ and renal cell carcinoma.^21^ Further evidence of the efficacy of durvalumab, either alone or combined with tremelimumab, is required from additional populations.

This phase I study investigated durvalumab monotherapy in patients from Japan with advanced solid tumors, and durvalumab monotherapy, and durvalumab plus tremelimumab combination therapy, in patients from Asia with BTC, ESCC, or HNSCC. In the preliminary dose escalation study of durvalumab monotherapy, the maximum tolerated dose was not reached, and dose‐limiting toxicities were not identified.^22^ For the dose expansion phase, a durvalumab dose of 10 mg/kg every 2 weeks (Q2W) was selected in the monotherapy cohort. For the combination cohort, durvalumab 20 mg/kg every 4 weeks (Q4W) plus tremelimumab 1 mg/kg Q4W for four doses, followed by durvalumab 20 mg/kg Q4W, was selected. This report presents the safety, tolerability, and antitumor activity of durvalumab as monotherapy, or in combination with tremelimumab, in patients with BTC, ESCC, or HNSCC from Japan, Taiwan, and the Republic of Korea.

## 2 METHODS

### 2.1 Study design and conduct

This was a phase I, open‐label, multicenter study (NCT01938612). During the previously published dose‐escalation phase, durvalumab monotherapy was evaluated in patients with advanced solid tumors.^22^ During the dose‐expansion phase, durvalumab monotherapy, and durvalumab plus tremelimumab were evaluated in patients with advanced BTC, ESCC, or HNSCC with disease progression during or following ≥1 platinum‐based therapy for advanced or metastatic disease.

For the study expansion phase, two regimens were selected. The first cohort of patients received durvalumab monotherapy: 10 mg/kg Q2W, via intravenous (IV) infusion, for either a maximum of 12 months or until confirmed progressive disease or unacceptable toxicity, initiation of an alternative cancer therapy, or withdrawal of consent. This dosing schedule for the durvalumab monotherapy cohort was determined by previous safety and efficacy data available for this dose. The durvalumab monotherapy cohort included three groups of patients with BTC, ESCC, or HNSCC. Approximately 20 to 60 patients were to be enrolled in each group. The second cohort received durvalumab and tremelimumab (durvalumab 20 mg/kg Q4W IV + tremelimumab 1 mg/kg Q4W IV for four doses) followed by durvalumab 20 mg/kg Q4W IV for either a maximum of 12 months or until confirmed progressive disease or unacceptable toxicity, initiation of an alternative cancer therapy, or withdrawal of consent. The dosing rationale for the combination cohort was based on data from another durvalumab plus tremelimumab study in solid tumors.^23^ The durvalumab plus tremelimumab cohort included two groups of patients with BTC or ESCC. Approximately 20 to 60 patients were to be enrolled in each group.

The protocol for this study (Study 2) was reviewed and approved by the appropriate review committees for each institution within which this work was undertaken. This study conforms to the provisions of the Declaration of Helsinki (as revised in Fortaleza, Brazil, October 2013). All patients were required to provide written informed consent prior to any study procedures.

### 2.2 Patient eligibility

Adults at least 20 years of age, with histologically or cytologically confirmed advanced or metastatic BTC, ESCC, or HNSCC, were eligible for enrollment. Patients with BTC or ESCC must have experienced disease progression during or following ≥1 platinum‐based therapy for unresectable disease for locally advanced, R/M disease. Patients with ESCC that demonstrated disease progression within 24 weeks of completing platinum‐containing therapy were enrolled. Patients with HNSCC must have demonstrated disease progression during or following ≥1 platinum‐based therapy for R/M disease. Eligible patients had ≥1 measurable lesion by Response Evaluation Criteria In Solid Tumors (RECIST) v1.1, an Eastern Cooperative Oncology Group (ECOG) performance status (PS) of 0 or 1, a minimum life expectancy of 16 weeks, and adequate organ and marrow function. Patients in the durvalumab monotherapy cohort were required to provide an unstained archived tumor tissue sample, as well as a recent tumor biopsy (taken following completion of the most recent therapy). The durvalumab plus tremelimumab cohort were to provide a fresh tumor biopsy at screening (preferred) or, alternatively, an archived tumor tissue sample obtained less than 3 years prior to screening. For both cohorts, the lesions for biopsy should not have been a RECIST target lesion, unless no others were suitable. If a RECIST target lesion was used, the longest diameter had to be ≥2 cm.

Exclusion criteria included treatment with any immunotherapy (including, but not restricted to, any anti‐PD‐1, anti‐PD‐L1, or anti‐CTLA‐4 antibody) or investigational anticancer treatment within 4 weeks prior to the initial dose of the study drug (within 6 weeks if mAb therapy). Other exclusion factors included: current or previous use of immunosuppressants within 28 days of the initial study drug dose (intranasal or inhaled corticosteroids or systemic corticosteroids at physiologic doses ≤ 10 mg/day of prednisolone were exempt); receipt of live attenuated vaccination within 30 days either preceding study enrollment or following receipt of study drug; major surgery within 30 days prior to the initial study drug dose, or undergoing recovery from previous surgery; and unresolved toxicity from previous anticancer treatment or any previous Grade ≥ 3 immune‐mediated adverse events (imAEs) while receiving immunotherapy. For the combination cohort, patients who experienced Grade ≥ 3 interstitial lung disease following any therapy were not enrolled. Additional exclusions included: any symptomatic or untreated central nervous system metastases requiring concurrent treatment; other invasive malignancy within 5 years preceding study enrollment; uncontrolled intercurrent illness; active or prior documented autoimmune disease within the last 2 years; history of primary immunodeficiency; or any condition that the investigators considered likely to interfere with assessment of the study drug.

### 2.3 Study assessments

The primary objective was to assess the safety and tolerability of durvalumab (±tremelimumab) in patients with advanced BTC, ESCC, or HNSCC. Secondary objectives were to evaluate the immunogenicity and antitumor activity of durvalumab (±tremelimumab). Analyses were performed by tumor type, with summary statistics reported for patient demographics and baseline characteristics, as well as safety data. PD‐L1 expression was measured from either archival or fresh (obtained at screening) tumor samples using the VENTANA PD‐L1 (SP263) Assay (Ventana Medical Systems, Inc.). PD‐L1 positive and high‐expression cutoffs evaluated were ≥1% tumor cells (TC ≥ 1%) or ≥25% tumor cells (TC ≥ 25%), respectively, at any staining intensity exceeding background.

All patients who received ≥1 dose of durvalumab per protocol were included in the safety analysis set. Survival data were analyzed using the full analysis set, and included every patient that received a dose of durvalumab before the data cutoff. Response data were assessed using the response evaluable set, and included every patient in the full analysis set that had measurable disease at baseline, as determined by the study investigators at a baseline disease assessment. AEs were analyzed using *National Cancer Institute Common Terminology Criteria for Adverse Events* v4.03 in the safety analysis. AEs were assessed by investigators; AE, serious AE (SAE), and concomitant treatment assessments were performed at regular intervals postdosing. AEs of special interest (AESI) were reviewed and imAEs were reported. An imAE was defined as an AESI that required treatment with systemic steroids or other immunosuppressants and/or endocrine therapy, was compatible with an immune‐mediated mechanism of action, and had no apparent alternative etiology. Serologic, immunologic, and histologic data, as required, were used to aid the characterization of imAEs. Antidrug antibody (ADA) analyses were conducted for durvalumab and tremelimumab; the ADA set included patients in all cohorts with available ADA data who received ≥ 1 dose of durvalumab.

## 3 RESULTS

### 3.1 Patients

The study data cutoff was March 31, 2018. Summary baseline demographics are reported in Table 1.

**TABLE 1:** Demographic characteristics (full analysis set)

		Durvalumab			Durvalumab + tremelimumab	
Characteristic		BTC (*n* = 42)	ESCC (*n* = 42)	HNSCC (*n* = 32)	BTC (*n* = 65)	ESCC (*n* = 59)
Age (years)	Median	64.0	63.0	62.0	62.0	62.0
	Min	37	45	33	28	42
	Max	81	74	73	78	77
Age group (years), *n* (%)	< 65 years, *n* (%)	22 (52.4)	26 (61.9)	19 (59.4)	43 (66.2)	33 (55.9)
	≥ 65 years, *n* (%)	20 (47.6)	16 (38.1)	13 (40.6)	22 (33.8)	26 (44.1)
Sex, *n* (%)	Male	24 (57.1)	36 (85.7)	28 (87.5)	43 (66.2)	56 (94.9)
	Female	18 (42.9)	6 (14.3)	4 (12.5)	22 (33.8)	3 (5.1)
Country, *n* (%)	Japan	42 (100.0)	35 (83.3)	32 (100.0)	40 (61.5)	29 (49.2)
	Republic of Korea	0	4 (9.5)	0	15 (23.1)	13 (22.0)
	Taiwan	0	3 (7.1)	0	10 (15.4)	17 (28.8)
Smoking status, *n* (%)	Non‐smoker	19 (45.2)	7 (16.7)	7 (21.9)	33 (50.8)	7 (11.9)
	Former smoker	19 (45.2)	32 (76.2)	23 (71.9)	29 (44.6)	44 (74.6)
	Current smoker	4 (9.5)	3 (7.1)	2 (6.3)	2 (3.1)	8 (13.6)
ECOG PS	0	27 (64.3)	22 (52.4)	15 (46.9)	32 (49.2)	26 (44.1)
	1	15 (35.7)	20 (47.6)	17 (53.1)	33 (50.8)	33 (55.9)
Previous lines of chemotherapy,^a^ *n* (%)	1	7 (16.7)	13 (31.0)	6 (18.8)	18 (27.7)	15 (25.4)
	2	24 (57.1)	14 (33.3)	12 (37.5)	24 (36.9)	16 (27.1)
	3	8 (19.0)	11 (26.2)	2 (6.3)	12 (18.5)	12 (20.3)
	≥ 4	3 (7.1)	4 (9.6)	12 (37.5)	11 (16.9)	16 (27.1)
Tumor PD‐L1 expression	TC < 1% PD‐L1^b^	13 (31.0)	10 (23.8)	7 (21.9)	35 (53.8)	16 (27.1)
	TC ≥ 1% PD‐L1^b^	19 (45.2)	25 (59.5)	19 (59.4)	18 (27.7)	33 (55.9)
	TC < 25% PD‐L1^c^	26 (61.9)	26 (61.9)	18 (56.3)	53 (81.5)	41 (69.5)
	TC ≥ 25% PD‐L1^c^	6 (14.3)	9 (21.4)	8 (25.0)	0	8 (13.6)

Abbreviations: BTC, biliary tract cancer; ECOG, Eastern Cooperative Oncology Group; ESCC, esophageal squamous cell carcinoma; HNSCC, head and neck squamous cell carcinoma; PD‐L1, programmed cell death ligand‐1; PS, performance status; TC, tumor cell.

^a^ Adjuvant and neoadjuvant therapies.

^b^ PD‐L1 high expression cutoff ≥ 1% tumor cells (TC ≥ 1%).

^c^ PD‐L1 high expression cutoff ≥ 25% tumor cells (TC ≥ 25%).

#### 3.1.1 Durvalumab monotherapy

In total, 120 patients were assigned durvalumab monotherapy (10 mg/kg Q2W). Of these, 116 patients received treatment. There were 42 patients with BTC, with tumor locations recorded as extrahepatic (*n* = 8, 19.0%), gall bladder (*n* = 19, 45.2%), intrahepatic (*n* = 13, 3.0%), or bile duct (*n* = 2, 4.8%). There were 42 patients with ESCC and 32 patients with HNSCC (Table 1). In the overall population, the median age was 63.5 years, 88 (75.9%) were male, and 109 (94.0%) were from Japan. Overall, most patients were current (*n* = 9, 7.8%) or former (*n* = 74, 63.8%) smokers (Table 1). Sixty‐four patients (55.2% of the overall population) had an ECOG PS of 0. However, in BTC, a greater number of patients had an ECOG PS of 0 (*n* = 27, 64.3%) than 1 (*n* = 15, 35.7%) (Table 1). More than two previous lines of therapy for metastatic disease had been received by 47 (40.5%) patients. A PD‐L1 expression of TC ≥ 1% was reported in 19 (45.2%), 25 (59.5%), and 19 (59.4%) patients with BTC, ESCC, and HNSCC, respectively (Table 1). At baseline, 6 (14.3%), 9 (21.4%), and 8 (25.0%) patients with BTC, ESCC, and HNSCC, respectively, had PD‐L1 expression of TC ≥ 25% (Table 1).

#### 3.1.2 Durvalumab plus tremelimumab

There were 127 patients assigned to durvalumab (20 mg/kg Q4W) plus tremelimumab (1 mg/kg Q4W), and 124 patients received treatment. The BTC group comprised 65 patients with tumor locations recorded as extrahepatic (*n* = 15, 23.1%), gall bladder (*n* = 16, 24.6%), intrahepatic (*n* = 31, 47.7%), ampulla of Vater (*n* = 2, 3.1%), and unknown (*n* = 1, 1.5%). There were 59 patients with ESCC (Table 1). The median age of the overall population was 62.0 years and 99 (79.8%) were male. Just over half of the patients (*n* = 69, 55.6%) were from Japan, 28 (22.6%) were from the Republic of Korea, and 27 (21.8%) were from Taiwan (Table 1). In the overall population, most patients were current (*n* = 10, 8.1%) or former (*n* = 73, 58.9%) smokers. An ECOG PS of 0 was recorded in 58 (46.8%) patients (Table 1) and 53 (42.7%) patients had received more than two previous lines of chemotherapy. At baseline, a PD‐L1 expression of TC ≥ 1% was recorded in 18 (27.7%) and 33 (55.9%) patients with BTC and ESCC, respectively (Table 1). Additionally, a PD‐L1 expression of TC ≥ 25% was recorded in 0 and 8 (13.6%) patients with BTC and ESCC, respectively (Table 1).

### 3.2 Safety

A summary of AE categories for both treatment groups (durvalumab monotherapy and durvalumab plus tremelimumab combination therapy) can be found in Table S1.

#### 3.2.1 Durvalumab monotherapy

Median (range) treatment duration for durvalumab monotherapy was 9.86 (1.3–52.1) weeks. Median (range) treatment duration was 11.8 (1.9–52.1) weeks in the BTC group, 9.8 (1.9–52.1) weeks in the ESCC group, and 8.9 (1.3–42.1) weeks in the HNSCC group. Overall, 103 (88.8%) patients experienced an AE and 31 (26.7%) were Grade ≥ 3 (Table S1). Treatment‐related AEs (TRAEs) of any grade were reported in 27 (64.3%) patients with BTC, 22 (52.4%) patients with ESCC, and 25 (78.1%) patients with HNSCC (Table 2). The most frequently reported TRAEs across all tumor types were fatigue, decreased appetite, diarrhea, stomatitis, and hypothyroidism (Table 2). Grade ≥ 3 TRAEs were reported in 8 (19.0%) patients with BTC, 4 (9.5%) patients with ESCC, and 8 (25.0%) patients with HNSCC. The most frequent were interstitial lung disease (BTC and HNSCC; *n* = 3 [2.6%]) and nausea (BTC and HNSCC), decreased appetite (ESCC and HNSCC), hyponatremia (BTC and ESCC), lymphocyte count decreased (BTC and HNSCC), and anemia (BTC) (*n* = 2 [1.7%] each). Treatment‐related SAEs were reported in 4 (9.5%) patients with BTC, 2 (4.8%) patients with ESCC, and 7 (21.9%) patients with HNSCC. Five patients in the monotherapy cohort discontinued therapy due to TRAEs (BTC, *n* = 2 [4.8%]; ESCC, *n* = 1 [2.4%]; HNSCC, *n* = 2 [6.3%]). In the monotherapy cohort, TRAEs with the outcome of death occurred in three patients; one in the ESCC group (gastrointestinal hemorrhage) and two in the HNSCC group (hemoptysis and hepatic function abnormal). Overall, there were 18 (15.5%) imAEs reported (BTC, *n* = 3 [7.1%]; ESCC, *n* = 8 [19.0%]; HNSCC, 7 [21.9%]) (Table S1). Treatments received and imAE outcomes are summarized in Table S1.

**TABLE 2:** Incidence of TRAEs (≥5% for any grade in at least one preferred term, in any arm and Grade ≥ 3) in the safety analysis set

	Durvalumab						Durvalumab + tremelimumab			
	BTC (*n* = 42)		ESCC (*n* = 42)		HNSCC (*n* = 32)		BTC (*n* = 65)		ESCC (*n* = 59)	
	Any grade	Grade ≥ 3	Any grade	Grade ≥ 3	Any grade	Grade ≥ 3	Any grade	Grade ≥ 3	Any grade	Grade ≥ 3
Patients with TRAE, *n* (%)	27 (64.3)	8 (19.0)	22 (52.4)	4 (9.5)	25 (78.1)	8 (25.0)	53 (81.5)	15 (23.1)	34 (57.6)	8 (13.6)
Adrenal insufficiency	0	0	0	0	0	0	4 (6.2)	3 (4.6)	2 (3.4)	1 (1.7)
Arthralgia	1 (2.4)	0	1 (2.4)	0	2 (6.3)	0	1 (1.5)	0	0	0
Decreased appetite	3 (7.1)	0	4 (9.5)	1 (2.4)	5 (15.6)	1 (3.1)	8 (12.3)	1 (1.5)	3 (5.1)	1 (1.7)
Diarrhea	4 (9.5)	0	4 (9.5)	0	3 (9.4)	0	8 (12.3)	0	6 (10.2)	1 (1.7)
Dry skin	1 (2.4)	1 (2.4)	0	0	3 (9.4)	0	5 (7.7)	0	0	0
Dysgeusia	2 (4.8)	0	0	0	2 (6.3)	0	0	0	2 (3.4)	0
Elevated AAT	0	0	1 (2.4)	0	1 (3.1)	0	2 (3.1)	0	3 (5.1)	0
Elevated ALT	0	0	1 (2.4)	0	1 (3.1)	0	2 (3.1)	0	3 (5.1)	0
Fatigue	4 (9.5)	0	4 (9.5)	0	4 (12.5)	1 (3.1)	7 (10.8)	1 (1.5)	0	0
Hepatic function abnormal	0	0	1 (2.4)	0	4 (12.5)	1 (3.1)	0	0	0	0
Hypertension	1 (2.4)	0	0	0	2 (6.3)	0	1 (1.5)	0	0	0
Hyperthyroidism	1 (2.4)	0	1 (2.4)	0	0	0	4 (6.2)	0	3 (5.1)	0
Hypoalbuminemia	3 (7.1)	0	0	0	0	0	0	0	0	0
Hypothyroidism	5 (11.9)	0	4 (9.5)	0	2 (6.3)	0	7 (10.8)	0	5 (8.5)	0
Infusion‐related reaction	2 (4.8)	0	3 (7.1)	0	0	0	0	0	3 (5.1)	0
Interstitial lung disease	2 (4.8)	1 (2.4)	0	0	2 (6.3)	2 (6.3)	1 (1.5)	0	3 (5.1)	1 (1.7)
Malaise	1 (2.4)	0	4 (9.5)	0	5 (15.6)	0	0	0	0	0
Nausea	2 (4.8)	1 (2.4)	2 (4.8)	0	5 (15.6)	1 (3.1)	4 (6.2)	1 (1.5)	2 (3.4)	0
Pyrexia	2 (4.8)	0	2 (4.8)	0	1 (3.1)	0	6 (9.2)	0	1 (1.7)	0
Pruritus	2 (4.8)	0	2 (4.8)	0	2 (6.3)	0	24 (36.9)	2 (3.1)	9 (15.3)	0
Rash	2 (4.8)	0	3 (7.1)	0	1 (3.1)	0	10 (15.4)	1 (1.5)	12 (20.3)	0
Rash maculo‐papular	1 (2.4)	0	0	0	4 (12.5)	0	4 (6.2)	1 (1.5)	0	0
Stomatitis	4 (9.5)	0	5 (11.9)	0	2 (6.3)	0	3 (4.6)	0	0	0
Vomiting	1 (2.4)	0	1 (2.4)	0	2 (6.3)	0	1 (1.5)	0	0	0

Abbreviations: ALT, alanine aminotransferase; AAT, aspartate aminotransferase; BTC, biliary tract cancer; ESCC, esophageal squamous cell carcinoma; TRAE, treatment‐related adverse event; HNSCC, head and neck squamous cell carcinoma.

#### 3.2.2 Durvalumab plus tremelimumab

The median (range) duration of exposure was 12.2 (0.7–53.9) weeks in the durvalumab plus tremelimumab cohort. Median (range) treatment duration was 11.9 (3.9–53.9) weeks for patients with BTC, and 15.7 (0.7–52.1) weeks for patients with ESCC. Overall, 117 (94.4%) patients experienced any AE and 61 (49.2%) were Grade ≥ 3 (Table S1). TRAEs were reported for 53 (81.5%) patients with BTC and 34 (57.6%) patients with ESCC. The most frequently reported TRAEs across tumor types were pruritus, rash, and diarrhea (Table 2). Grade ≥ 3 TRAEs were reported in 15 (23.1%) patients with BTC and 8 (13.6%) patients with ESCC. The most frequent (≥2 patients in BTC and ESCC) were adrenal insufficiency (*n* = 4 [3.2%]), and decreased appetite, hyponatremia, and pruritus (≥2 patients BTC only) (*n* = 2 [1.6%] each). Treatment‐related SAEs were reported in 9 (13.8%) patients with BTC and 8 (13.6%) patients with ESCC. Eight patients in the combination cohort discontinued because of TRAEs (BTC, *n* = 5 [7.7%]; ESCC, *n* = 3 [5.1%]). TRAEs with the outcome of death occurred in one patient with BTC who experienced drug‐induced liver injury. Overall, there were 32 (25.8%) imAEs reported (BTC, *n* = 15 [23.1%]; ESCC, *n* = 11 [32.8%]) (Table S1). Treatments received and imAE outcomes are summarized in Table S1.

### 3.3 Immunogenicity

#### 3.3.1 Durvalumab monotherapy

Among the 112 patients with baseline ADA results, there were two patients with ADA to durvalumab at baseline; 1 (2.4%) patient with BTC and 1 (3.4%) with HNSCC. Neither of these patients showed evidence of ADA post baseline. Post baseline, there were 9 (8.0%) patients who were ADA positive to durvalumab (BTC, *n* = 6 [14.3%]; ESCC, *n* = 2 [4.9%]; HNSCC, *n* = 1 [3.4%]). In the BTC group, 2 (4.8%) patients were transient positive. None of the patients who were ADA positive post baseline had neutralizing antibodies (nAb) post baseline (Table S2).

#### 3.3.2 Durvalumab plus tremelimumab

Of the 102 patients with baseline ADA results, three (5.4%) patients with BTC had positive ADA to durvalumab at baseline. None of the patients with ESCC were ADA positive at baseline. Post baseline, there were four (3.9%) patients who were ADA positive (BTC, *n* = 3 [5.4%], ESCC, *n* = 1 [2.2%]). There were no transient positive results. The one (2.2%) patient with ESCC who was ADA positive post baseline was also nAb positive (Table S2).

For ADAs to tremelimumab (*n* = 102), there were three (5.4%) patients in the BTC group who were ADA positive at baseline and none in the ESCC group. Post baseline, there were 16 (17%) patients who were ADA positive (BTC, *n* = 10 [20.8%], ESCC, *n* = 6 [13%]). In addition, of those who were ADA positive post baseline, 10 (20.8%) patients in the BTC group and five (10.9%) patients in the ESCC group were nAb positive post baseline (Table S2).

### 3.4 Efficacy

#### 3.4.1 Durvalumab monotherapy

The objective response rate (ORR; investigator assessed) was 4.8% (2 of 42 patients) for patients with BTC, 7.1% (3 out of 42 patients) for patients with ESCC, and 9.4% (3 out of 32 patients) for patients with HNSCC (Table 3). All observed responses were partial responses. There were no complete responses. The median duration of response (DoR) was 9.7 months in patients with BTC, 2.5 months in patients with ESCC, and 5.1 months in patients with HNSCC (Table 3). Where samples with tumor quality sufficient to detect the PD‐L1 status were available, in ESCC or HNSCC, ORR was greater for patients with PD‐L1 expression versus no expression using the TC ≥ 1% cutoff (Table 3). Positive PD‐L1 expression (TC ≥ 1%), was not linked to a greater ORR for patients with BTC. In all three groups, the ORR in patients with high expression (PD‐L1 TC ≥ 25%) was higher than the patients with low‐PD‐L1 expression (TC < 25%) (Table 3).

**TABLE 3:** Best overall response across treatment and PD‐L1 groups

	Durvalumab			Durvalumab + tremelimumab	
Best overall response	BTC (*n* = 42)	ESCC (*n* = 42)	HNSCC (*n* = 32)	BTC (*n* = 65)	ESCC (*n* = 59)
ORR, *n* (%) [95% CI]	2 (4.8) [0.6–16.1]	3 (7.1) [1.5–19.4]	3 (9.4) [2.0–25.0]	7 (10.8) [6.1–17.5]	12 (20.3) [11.0–32.8]
CR, *n*	0	0	0	0	2
PR, *n*	2	3	3	7	10
Median DoR, months	9.7	2.5	5.1	8.4	19.6
PD‐L1 TC < 1%^a^	*n* = 13	*n* = 10	*n* = 7	*n* = 35	*n* = 16
ORR, *n* (%) [95% CI]	1 (7.7) [0.2–36.0]	0 (0.0) [0.0–30.9]	0 (0.0) [0.0–41.0]	4 (11.4) [3.2–26.7]	1 (6.3) [0.2–30.2)
PD‐L1 TC ≥ 1%^a^	*n* = 19	*n* = 25	*n* = 19	*n* = 18	*n* = 33
ORR, *n* (%) [95% CI]	1 (5.3) [0.1–26.0]	3 (12.0) [2.6–31.2]	3 (15.8) [3.4–39.6]	1 (5.6) [0.1–27.3]	8 (24.2) [11.1–42.3]
PD‐L1 TC < 25%^b^	*n* = 26	*n* = 26	*n* = 18	*n* = 53	*n* = 41
ORR, *n* (%) [95% CI]	1 (3.9) [0.1–19.6]	1 (3.9) [0.1–19.6]	1 (5.6) [0.1–27.3]	5 (9.4) [3.1–20.7]	5 (12.2) [4.1–26.2]
PD‐L1 TC ≥ 25%^b^	*n* = 6	*n* = 9	*n* = 8	*n* = 0	*n* = 8
ORR, *n* (%) [95% CI]	1 (16.7) [0.4–64.1]	2 (22.2) [2.8–60.0]	2 (25.0) [3.1–65.1]	NA	4 (50.0) [15.7–84.3]

Abbreviations: BTC, biliary tract cancer; CR, complete response; DoR, duration of response; ESCC, esophageal squamous cell carcinoma; HNSCC, head and neck squamous cell carcinoma; NA, not applicable; ORR, objective response rate; PD‐L1, programmed cell death ligand‐1; PR, partial response; TC, tumor cell.

^a^ PD‐L1 high expression cutoff ≥ 1% tumor cells (TC ≥ 1%).

^b^ PD‐L1 high expression cutoff ≥ 25% tumor cells (TC ≥ 25%).

**FIGURE 1:**
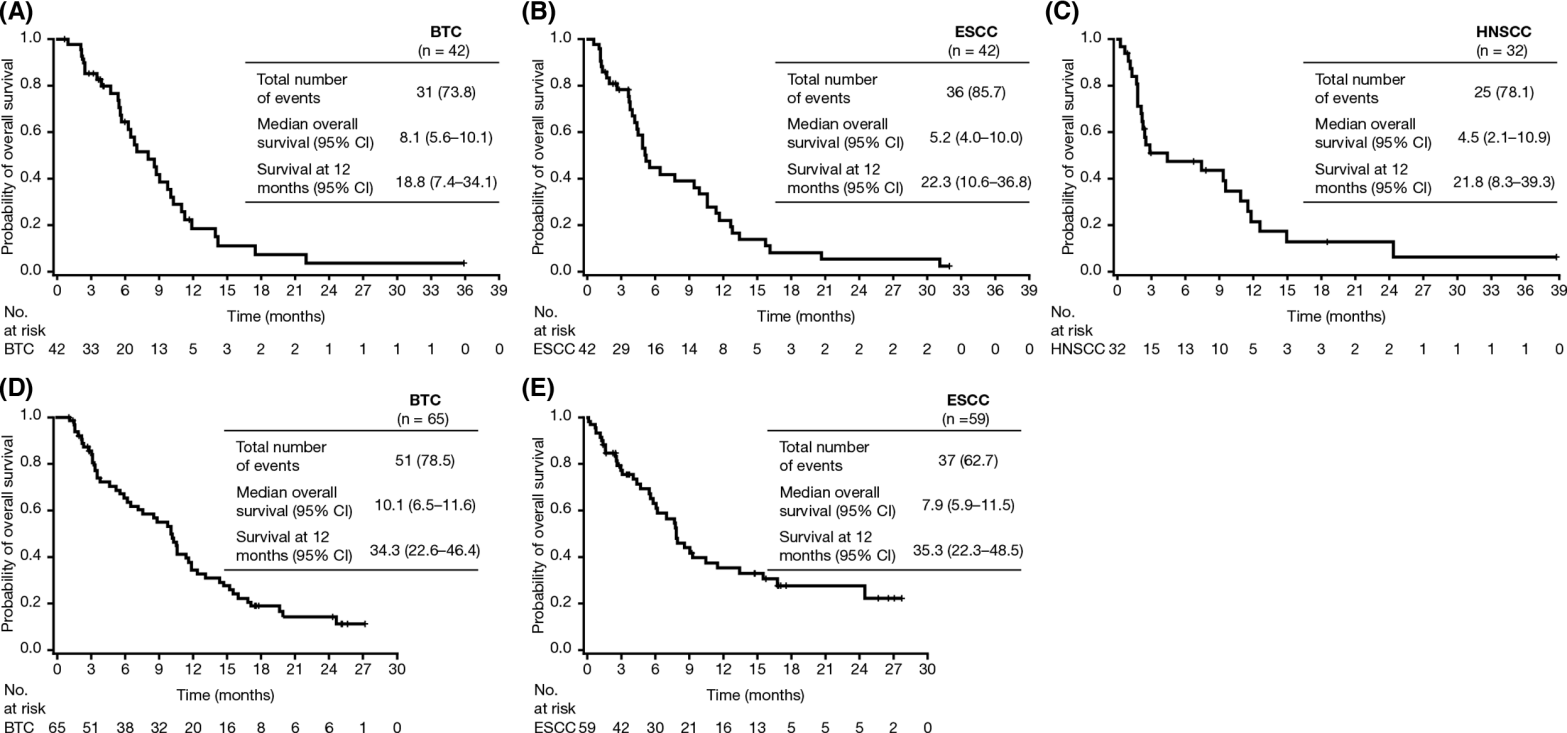
Overall survival in patients with BTC, ESCC, or HNSCC in the durvalumab monotherapy and durvalumab plus tremelimumab cohorts. Durvalumab monotherapy in patients with (A) BTC, (B) ESCC, and (C) HNSCC, and durvalumab plus tremelimumab in patients with (D) BTC and (E) ESCC. BTC, biliary tract cancer; ESCC, esophageal squamous cell carcinoma; HNSCC, head and neck squamous cell carcinoma

Median progression‐free survival (PFS) was 1.5 months (95% confidence interval [CI] 1.4–2.6) in patients with BTC, 1.4 months (95% CI 1.3–1.6) in patients with ESCC, and 1.4 months (95% CI 1.1–1.6) in patients with HNSCC. Median overall survival (OS) was 8.1 months (95% CI 5.6–10.1) in patients with BTC, 5.2 months (95% CI 4.0–10.0) in patients with ESCC, and 4.5 months (95% CI 2.1–10.9) in patients with HNSCC (Figure 1A–C). OS at 12 months was 18.8% (95% CI 7.4–34.1), 22.3% (95% CI 10.6–36.8), and 21.8% (95% CI 8.3–39.3) for patients with BTC, ESCC, and HNSCC, respectively.

#### 3.4.2 Durvalumab plus tremelimumab

For patients with BTC, the ORR (investigator assessed) was 10.8% (7 of 65 patients) and 20.3% (12 of 59 patients) in patients with ESCC. Complete response was experienced by two patients with ESCC, with 10 partial responses. In the BTC group, there were seven partial responses and no complete responses (Table 3). The median DoR was 8.4 months in patients with BTC and 19.6 months in patients with ESCC (Table 3). In the ESCC group, the ORR in the PD‐L1 TC ≥ 25% group was higher (50.0%) compared with the PD‐L1 TC < 25% group (12.2%) (Table 3). Using the PD‐L1 TC 1% cutoff, PD‐L1 positive expression (TC ≥ 1%) was linked to a higher ORR versus no expression (TC < 1%) in ESCC (24.2% vs. 6.3%) but not for BTC (5.6% vs. 11.4%) (Table 3).

Median PFS was 1.6 months (95% CI 1.4–2.8) in patients with BTC and 2.9 months (95% CI 1.6–3.5) in patients with ESCC. Median OS was 10.1 months (95% CI 6.5–11.6) in the BTC group and 7.9 months (95% CI 5.9–11.5) in the ESCC group (Figure 1D,E). OS at 12 months was 34.3% (95% CI 22.6–46.4) and 35.3% (95% CI 22.3–48.5) for BTC and ESCC, respectively.

## 4 DISCUSSION

In this phase I study, durvalumab monotherapy and durvalumab plus tremelimumab both displayed an acceptable safety profile and preliminary clinical activity in patients with BTC, ESCC, or HNSCC.

In general, the safety profile for durvalumab monotherapy was consistent with published literature. However, the percentage of TRAEs Grade ≥ 3 in the HNSCC group in this study (25%) appeared higher than previously reported in the literature^14^, ^15^, ^16^ with TRAEs Grade ≥ 3 reported at between 8%^16^ and 12.3%^14^ for durvalumab monotherapy in HNSCC. The reason for the higher rate of AEs observed is unclear, but in a small sample size it is possible to have fluctuations in numbers of AEs reported. Despite the higher rate of TRAEs in the HNSCC durvalumab monotherapy group, only two (6.2%) patients discontinued treatment as a result of therapy, suggesting the events were manageable. Some TRAEs were reported more frequently in the durvalumab plus tremelimumab cohort versus the durvalumab monotherapy cohort, including pruritus and rash, which is consistent with the literature where a higher incidence of AEs and high‐grade AEs are reported in anti‐PD‐1/PD‐L1 and anti‐CTLA‐4 combination therapies.

The incidence of positive ADA or nAb to durvalumab in this study was low and in line with previously reported data.^24^ Clinical activity was observed in both cohorts (durvalumab monotherapy and durvalumab plus tremelimumab), and although this phase I study was not designed to make comparisons between monotherapy and combination therapy, or across cancer types, efficacy appeared to be numerically higher for the patients with BTC and ESCC included in the durvalumab plus tremelimumab cohort. In BTC, the ORR and OS in the durvalumab plus tremelimumab cohort compare favorably with published data on chemotherapy^25^ and immunotherapy (second‐ and third‐line settings),^10^ and provides support for further development of immunotherapy combinations in BTC.

As with other checkpoint inhibitors such as pembrolizumab and nivolumab,^26^, ^27^ which have shown activity in randomized controlled trials in the second‐line setting, durvalumab showed clear signs of activity with durable responses in ESCC. Subtle differences in ORR between trials may be explained, in part, by differences in patient characteristics. For ESCC the ORR for durvalumab monotherapy, 7.1% (95% CI 1.5–19.4) was numerically lower than that for nivolumab, 19% (95 CI 14–26)^26^ and for pembrolizumab, 14.3% (95% CI 6.7–25.4).^27^ This is likely owing to the cohort of patients enrolled in this study who were heavily pretreated and who had a poor prognosis. The ORR reported in patients with ESCC in the durvalumab plus tremelimumab group (20.3%; 95% CI 11.0–32.8) was higher than that in the monotherapy group (7.1%; 95% CI 1.5–19.4) with complete response reported in two patients. Of interest, the survival curve in patients with ESCC in the durvalumab plus tremelimumab cohort appears to plateau at around 20%–25% beyond 2 years, indicating that a subgroup of individuals may obtain long‐term benefit from this treatment. The response rates in this study support the application of immune checkpoint inhibitors as monotherapy or combination therapy as important treatment strategies for patients with ESCC.

PD‐L1 expression on tumor cells and immune cells has been linked to greater clinical benefit from anti‐PD‐1‐ and anti‐PD‐L1‐targeted therapies across multiple tumor types. In the durvalumab monotherapy cohort in this study, ORR was numerically higher for all cancer types in patients with PD‐L1 expression using both PD‐L1 cutoffs (TC ≥ 25% and TC ≥ 1%) than in patients with PD‐L1 low or no expression (TC < 25% or TC < 1%), with the exception of BTC, which showed a higher ORR using the PD‐L1 cutoff of TC ≥ 25% but not with TC ≥ 1%. For the durvalumab plus tremelimumab cohort, the ORR was also higher in patients with PD‐L1 expression (TC ≥ 25% and TC ≥ 1%) in patients with ESCC. This trend was not confirmed in patients with BTC, since TC ≥ 1% was not associated with a higher ORR and none of the patients met the criteria for PD‐L1 TC ≥ 25%. The general trend for a higher ORR in PD‐L1 positive (TC ≥ 25% and TC ≥ 1%) patients with ESCC and HNSCC is consistent with published reports regardless of tumor type; however, any potential trends should be interpreted with caution due to the limited sample size in the PD‐L1 evaluable population.

In conclusion, the safety profiles and the preliminary efficacy of durvalumab in combination with tremelimumab revealed through this study support further clinical development of anti‐PD‐1/PD‐L1 and anti‐CTLA‐4 combinations in additional tumor types. Durvalumab monotherapy and durvalumab plus tremelimumab both demonstrated acceptable toxicity profiles and clinical benefit in patients from Asia with BTC, ESCC, and HNSCC who had progressed on previous systemic chemotherapy.

## CONFLICT OF INTEREST

Yuichiro Doki: Personal fees from Ono pharmaceutical company, outside the submitted work. Makoto Ueno: Grants and personal fees from AstraZeneca, during the conduct of the study. Grants and/or personal fees from Astellas Pharma, Daiichi Sankyo, Dainippon Sumitomo Pharma, Eisai, Incyte, Merck Serono, MSD, Nipro Corporation, Ono Pharmaceutical, Taiho Pharmaceutical, Teijin Pharma, and Yakult Honsha, outside the submitted work. Chih‐Hung Hsu: Grants from AstraZeneca, during the conduct of the study; Grants and/or personal fees from Beigene, Bristol‐Myers Squibb, Genentech, Merck Serono, Merck Sharp & Dohme, Ono Pharmaceutical, and Roche, outside the submitted work. Do‐Youn Oh: Nothing to disclose. Keunchil Park: Personal fees and other support from AstraZeneca, outside the submitted work. Noboru Yamamoto: Grants from Astellas, Bayer, Boehringer Ingelheim, Bristol‐Myers Squibb, Chugai, Daiichi‐Sankyo, Eisai, Kyowa‐Hakko Kirin, Lilly, Novartis, Ono Pharmaceutical, Pfizer, Quintiles, Takeda and Taiho. Personal fees from AstraZeneca, Boehringer Ingelheim, Bristol‐Myers Squibb, Chugai, Cimic, Eisai, Lilly, Ono Pharmaceutical, Otsuka, Pfizer, Sysmex, and Takeda. Grants from Chiome Bioscience Inc., GSK, Janssen Pharma, Merck, MSD, and Sumitomo Dainippon, outside the submitted work. Tatsuya Ioka: Grants from AstraZeneca, during the conduct of the study. Grants from Dainippon Sumitomo, Eisai, Incyte, Shire, Taiho, and Takara Bio; Personal fees from Chugai, Servier, Taiho, and Yakult Honsha, outside the submitted work. Hiroki Hara: Grants and/or personal fees from Astellas, AstraZeneca, Beigene, Boehringer Ingelheim, Bristol‐Myers Squibb, Chugai, Daiichi Sankyo, Dainippon Sumitomo Pharma, Eisai, Incyte, Kyowa Hakko Kirin, Lilly, LSK BioPharma, Merck Biopharma, MSD, Ono Pharmaceutical, Pfizer, Sanofi, Taiho, Takeda, and Yakult Honsha, outside the submitted work. Manabu Hayama: Is an employee of AstraZeneca. Masahiro Nii: Is an employee of AstraZeneca. Keiko Komuro: Is an employee of AstraZeneca. Mariko Sugimoto: Is an employee of AstraZeneca. Makoto Tahara: Grants and personal fees from AstraZeneca, during the conduct of the study. Grants and/or personal fees from Amgen, Bayer, Bristol‐Myers Squibb, Celgene, Eisai, LOXO, Merck Serono, MSD, Novartis, Ono Pharmaceutical, Pfizer, and Rakuten Medical, outside the submitted work.

## AUTHOR CONTRIBUTIONS

Yuichiro Doki: Resources, investigation, and writing– original draft, review and editing. Makoto Ueno: Resources, investigation, and writing– original draft, review and editing. Chih‐Hung Hsu: Resources, investigation, and writing– original draft, review and editing. Do‐Youn Oh: Resources, investigation, and writing– original draft, review and editing. Keunchil Park: Resources, investigation, and writing– original draft, review and editing. Noboru Yamamoto: Resources, investigation, and writing– original draft, review and editing. Tatsuya Ioka: Resources, investigation, and writing– original draft, review and editing. Hiroki Hara: Resources, investigation, and writing– original draft, review and editing. Manabu Hayama: Data curation and writing– original draft, review and editing. Masahiro Nii: Conceptualization, methodology, data curation, formal analysis, and writing– original draft, review and editing. Keiko Komuro: Data curation and writing– original draft, review and editing. Mariko Sugimoto: Data curation and writing– original draft, review and editing. Makoto Tahara: Resources, investigation, and writing– original draft, review and editing.

## ETHICS STATEMENT

The protocol for this study (Study 2) was reviewed and approved by the appropriate review committees for each institution within which this work was undertaken. This study conforms to the provisions of the Declaration of Helsinki (as revised in Fortaleza, Brazil, October 2013). All patients were required to provide written informed consent prior to any study procedures.

## Supporting information


Table S1
Table S2

## Data Availability

Data underlying the findings described in this manuscript may be obtained in accordance with AstraZeneca’s data sharing policy described at: https://astrazenecagrouptrials.pharmacm.com/ST/Submission/Disclosure
