# Supplementary material for: Tolerability and efficacy of durvalumab, either as monotherapy or in combination with tremelimumab, in patients from Asia with advanced biliary tract, esophageal, or head‐and‐neck cancer
Source: Cancer Med. 2022 May 24;11(13):2550–60. doi: 10.1002/cam4.4593 (PMC9249982; doi:10.1002/cam4.4593)
Supplement: Supplementary file 1 — Table S1 Table S2 [file CAM4-11-2550-s001.docx]

# Supplementary Information

## Supplementary TABLE 1 Summary of adverse events categories

|  | Durvalumab monotherapy | | | Durvalumab + tremelimumab | |
| --- | --- | --- | --- | --- | --- |
|  | **BTC (n = 42)** | **ESCC (n = 42)** | **HNSCC (n = 32)** | **BTC (n = 65)** | **ESCC (n = 59)** |
| Any AE^†^ | 36 (85.7) | 36 (85.7) | 31 (96.9) | 61 (93.8) | 56 (94.9) |
| Any AE grade ≥3 | 9 (21.4) | 8 (19.0) | 14 (43.8) | 32 (49.2) | 29 (49.2) |
| Any AE with outcome of death | 0 | 1 (2.4) | 3 (9.4) | 2 (3.1) | 3 (5.1) |
| Any AE leading to discontinuation of study treatment | 2 (4.8) | 2 (4.8) | 2 (6.3) | 6 (9.2) | 5 (8.5) |
| Treatment-related AE^‡^ | 27 (64.3) | 22 (52.4) | 25 (78.1) | 53 (81.5) | 34 (57.6) |
| Treatment-related AE grade ≥3^‡^ | 8 (19.0) | 4 (9.5) | 8 (25.0) | 15 (23.1) | 8 (13.6) |
| Treatment related AE with outcome of death^‡^ | 0 | 1 (2.4) | 2 (6.3) | 1 (1.5) | 0 |
| Treatment-related AE leading to discontinuation of study treatment^‡^ | 2 (4.8) | 1 (2.4) | 2 (6.3) | 5 (7.7) | 3 (5.1) |
| Any SAE^§^ | 4 (9.5) | 7 (16.7) | 10 (31.3) | 22 (33.8) | 28 (47.5) |
| Treatment-related SAE^‡,§^ | 4 (9.5) | 2 (4.8) | 7 (21.9) | 9 (13.8) | 8 (13.6) |
| Any AESI | 19 (45.2) | 15 (35.7) | 20 (62.5) | 50 (76.9) | 38 (64.4) |
| Any AESI grade ≥3 | 2 (4.8) | 2 (4.8) | 3 (9.4) | 14 (21.5) | 6 (10.2) |
| Treatment-related AESI | 15 (35.7) | 12 (28.6) | 16 (50.0) | 46 (70.8) | 29 (49.2) |
| Any imAEs | 3 (7.1) | 8 (19.0) | 7 (21.9) | 15 (23.1) | 17 (28.8) |
| imAEs ≥3 | 2 (4.8) | 0 | 2 (6.3) | 5 (7.7) | 5 (8.5) |
| imAEs with outcome of death | 0 | 0 | 1 (3.1) | 0 | 0 |
| imAEs leading to discontinuation of study treatment | 1 (2.4) | 0 | 2 (6.3) | 1 (1.5) | 3 (5.1) |
| Received systemic corticosteroids | 3 (7.1) | 6 (14.3) | 5 (15.6) | 11 (16.9) | 13 (22.0) |
| Received high-dose steroids | 2 (4.8) | 3 (7.1) | 3 (9.4) | 7 (10.8) | 7 (11.9) |
| Received endocrine therapy | 1 (2.4) | 4 (9.5) | 2 (6.3) | 6 (9.2) | 6 (10.2) |
| imAE outcome resolved | 0 | 4 (9.5) | 2 (6.3) | 2 (3.1) | 7 (11.9) |

^†^Patients with multiple events in the same category are counted only once in that category. Patients with events in more than one category are counted once in each of those categories. ^‡^As assessed by the investigator. ^§^Including events with outcome = death. Abbreviations: AESI, adverse events of special interest; BTC, biliary tract carcinoma; ESCC, esophageal squamous cell carcinoma; HNSCC, squamous cell carcinoma of the head and neck; imAEs, immune-mediated adverse events; SAE, serious adverse event.

## Supplemental TABLE 2 Antidrug antibody (ADA) response to durvalumab and tremelimumab

|  | Durvalumab | | | Durvalumab + tremelimumab | | | |  |
| --- | --- | --- | --- | --- | --- | --- | --- | --- |
|  | Durvalumab | | | Durvalumab | | Tremelimumab | |  |
|  | **BTC**  (n = 42) | **ESCC** (n = 41) | HNSCC  (n = 29) | **BTC** (n = 56) | **ESCC** (n = 46) | **BTC** (n = 56) | **ESCC**  (n = 46) |  |
| Baseline ADA results n (%) | 41 (97.6) | 41 (100.0) | 29 (100.0) | 56 (100.0) | 46 (100.0) | 56 (100.0) | 46 (100.0) | |
| ADA positive | 1 (2.4) | 0 | 1 (3.4) | 3 (5.4) | 0 | 3 (5.4) | 0 | |
| Postbaseline ADA results | 42 (100.0) | 41 (100.0) | 29 (100.0) | 56 (100.0) | 46 (100.0) | 48 (85.7) | 46 (100.0) | |
| ADA positive | 6 (14.3) | 2 (4.9) | 1 (3.4) | 3 (5.4) | 1 (2.2) | 10 (20.8) | 6 (13.0) | |
| Treatment boosted^†^ | 0 | 0 | 0 | 0 | 0 | 0 | 0 | |
| Persistent positive^‡^ | 4 (9.5) | 2 (4.9) | 1 (3.4) | 2 (3.6) | 1 (2.2) | 8 (16.7) | 5 (10.9) | |
| Transient positive^§^ | 2 (4.8) | 0 | 0 | 0 | 0 | 1 (2.1) | 1 (2.2) | |
| nAb positive | 0 | 0 | 0 | 0 | 1 (2.2) | 10 (20.8) | 1. (10.9) | |

^†^Positive ADA titer that was boosted to a 4-fold or higher-level following treatment. ^‡^Positive at ≥2 postbaseline assessments (with ≥16 weeks between first and last positive) or positive at last postbaseline assessment. ^§^At least one postbaseline ADA positive assessment but not fulfilling the conditions of persistently positive. Abbreviations: BTC, biliary tract carcinoma; ESCC, esophageal squamous cell carcinoma; HNSCC, squamous cell carcinoma of the head and neck.
